# Predicting difficult airway in morbidly obese patients using ultrasound

**DOI:** 10.55730/1300-0144.5787

**Published:** 2023-11-21

**Authors:** Sevim AKIN, Mustafa YILDIRIM, Hakan ARTAŞ, Esef BOLAT

**Affiliations:** 1 Department of Anesthesiology and Reanimation, Faculty of Medicine, Fırat University, Elazığ, Turkiye; 2 Department of Radiology, Faculty of Medicine, Fırat University, Elazığ, Turkiye

**Keywords:** Obesity, difficult airway, ultrasound, intubation, neck

## Abstract

**Background/aim:**

Difficult mask ventilation and difficult intubation are more common in obese patients. Ultrasound is a reliable and noninvasive method for evaluating the airway. The aim of this study was to investigate the contribution and availability of anterior neck soft tissue (ANS) thickness at different levels, tongue volume (TV), hyomental distance (HMD), the ratio of preepiglottic distance to distance between the epiglottis and the midpoint of vocal cords (PE/E-VC) measured by ultrasonography in predicting difficult airway in morbidly obese patients.

**Materials and methods:**

Between March 2020 and November 2020, patients aged ≥18 years with a body mass index (BMI) of ≥40 kg/m2 who underwent elective surgery under general anesthesia were included in this prospective study at Fırat University Hospital. During the preoperative evaluation of patients, ultrasound was used to measure and record TV, ANS thickness at different levels, HMD, and ratio of PE/E-VC. Patients with difficult intubation were identified using the Cormack-Lehane classification system. Patients whohad difficulties with balloon mask ventilation were recorded. Subsequently, the parameters of patients with easy and difficult intubation were compared. In addition, the parameters of patients with easy and difficult mask ventilation were also compared.

**Results:**

The preepiglottic ANS thickness at the level of the thyrohyoid membrane and the PE/E-VC value in obese patients with difficult intubation were significantly greater than in obese patients with easy intubation (p < 0.001). In addition, TV (p < 0.001), preepiglottic ANS thickness at the thyrohyoid membrane level (p < 0.001), ANS thickness at the thyroid isthmus level (p = 0.002), ANS-suprasternal notch thickness (p = 0.004), and PE/E-VC (p = 0.005) values were significantly greater in obese patients with difficult mask ventilation.

**Conclusion:**

Ultrasound may be a useful tool for predicting difficult airway and difficult mask ventilation. For this purpose, ANS thickness at different levels, PE/E-VC, and TV values measured by ultrasound can be used.

## 1.Introduction

Difficult airway is the main cause of anesthesia-related mortality and morbidity, particularly in emergency cases [1]. Failure to successfully manage a difficult airway may be the main cause of 30% of anesthesia-related deaths [2]. According to the 2022 ASA (American Society of Anesthesiologists) guidelines, a difficult airway is any clinical condition in which a doctor with training in anesthetic treatment encounters anticipated or unexpected difficulties or failure, including one or more of the following: laryngoscopy, mask ventilation, ventilation with supraglottic airway, tracheal intubation, invasive airway, or extubation [3]. Difficult tracheal intubation was defined in the 2022 ASA guideline as a condition where multiple attempts are required for successful tracheal intubation, or when it remains unsuccessful after several trials [3].

Obesity poses a significant public health problem, characterized by an increasing incidence [4]. Obese patients frequently experience difficulties in both mask ventilation and intubation [5]. The frequency of difficult intubation is between 0.1% and 13% in the normal population; however, this rate can reach up to 14% in obese patients [6]. In a metaanalysis, it was reported that difficult intubation occurred three times more frequently in morbidly obese patients than in those with normal weight [7].

The risk of brain damage and death increases with difficulty in airway patency. Therefore, the predetermination and appropriate management of difficult airway has an important role in preventing anesthesia-related mortality and morbidity. Predicting difficult intubation allows for modification of the anesthetic method and the availability of assistive equipment and personnel if intubation is to be performed. This may result in a reduction in the risk of developing complications. There are several clinical determinants for difficult airway intubation, including Mallampati score, sternomental distance, thyromental distance, short neck, and neck circumference. The Mallampati classification is based on the appearance of the tongue base and pharyngeal structures [8]. However, none of these methods has been proven to be reliable on its own. Cormack-Lehane (C-L) grading is determined based on the appearance of the vocal cords and epiglottis during direct laryngoscopy. Its contribution to difficult airway preparation is limited because the patient is evaluated by laryngoscopy after the induction of general anesthesia on the operating table. As the tests used for predicting difficult airway are at times insufficient, the search for predictive tests, which are suitable for practical use, characterized by high reliability and ease of application, continues [9].

Although obesity is assumed to increase the likelihood of difficult intubation, a higher body mass index is not reliable criteria for predicting difficult laryngoscopy [10]. In contrast to body mass index, a larger neck circumference is a stronger indicator of difficult laryngoscopy [6]. Nevertheless, fat distribution in specific neck regions may be more useful than neck circumference in predicting difficult airway. The ultrasonographic imaging technique has recently emerged as an important tool in the imaging and evaluation of the airway, owing to its simplicity, noninvasiveness, and portability [11]. According to our hypothesis, measuring the amount of soft tissue at different locations using ultrasound may be useful in estimating difficult airway.

The aim of this study was to investigate the contribution and availability of anterior neck soft tissue (ANS) thickness, tongue volume (TV), hyomental distance (HMD), the ratio of preepiglottic distance to distance between the epiglottis and the midpoint of vocal cords (PE/E-VC) in estimating difficult airway among morbidly obese patients.

## 2. Materials and methods

**Figure 1:**
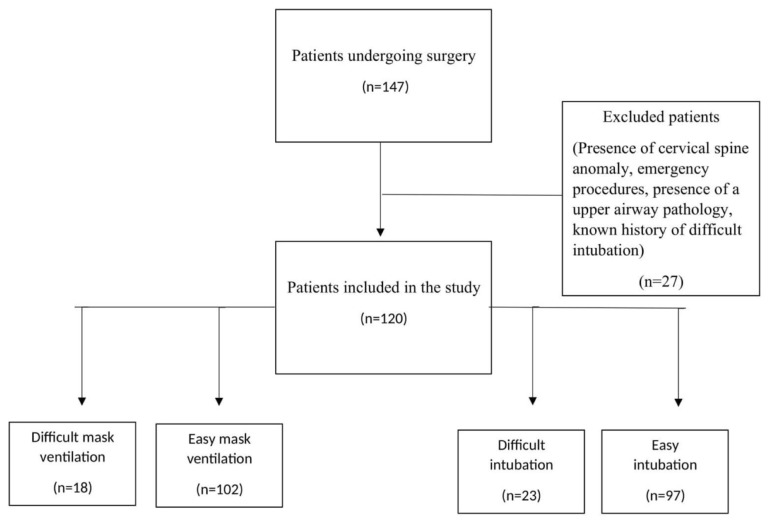
A flow chart of patients included in the study.

Ethic approval was obtained from the local ethics committee for this prospective study (date: 28.02.2020, no: 381516). This study was conducted with obese patients scheduled for elective surgery involving endotracheal intubation under general anesthesia and in the sniffing position between March 1, 2020 and November 1, 2020 at Fırat University Hospital. Power analysis was performed using G*Power software to determine the number of patients in the study, and written informed consent was obtained from all patients. Inclusion criteria are as follows: 1) General anesthesia with endotracheal intubation, 2) elective surgery, 3) age ≥18 years, 4) body mass index (BMI) ≥40 kg/m^2^. Exclusion criteria are as follows: 1) presence of cervical spine anomaly, 2) emergency procedures, 3) presence of a pathology that causes anatomical changes in the upper airway (such as maxillo-facial fractures, previous surgery, or tumor), 4) known history of difficult intubation (Figure 1). Additionally, all patients were tested for coronavirus disease 2019 (COVID-19) within 24 h before the operation.

**Figure 2:**
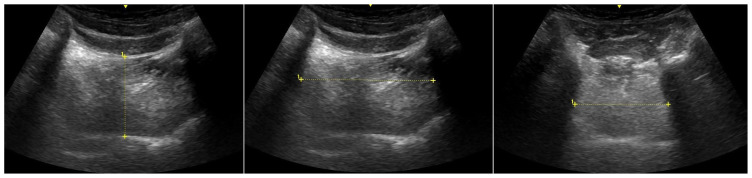
Three dimensions of the tongue and tongue volume measurement.

**Figure 3:**
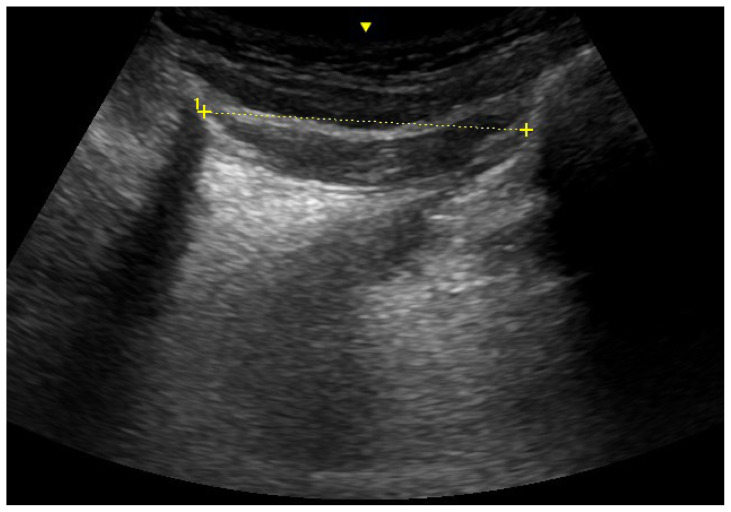
Sonographic measurement of hyomental distance.

**Figure 4:**
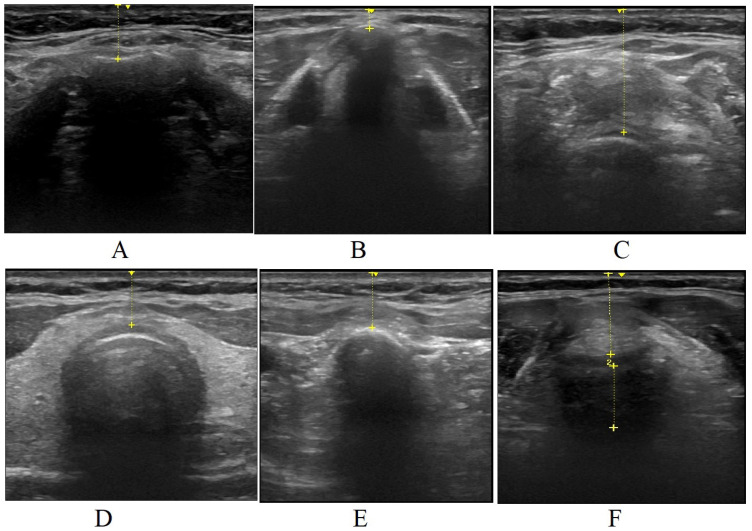
ANS thickness levels. A) ANS thickness at hyoid bone level. B) ANS thickness at vocal cord level. C) Preepiglottic ANS thickness at thyrohyoid membrane level. D) ANS thickness at isthmus level. E) ANS thickness at suprasternal notch level. F) Preepiglottic distance to distance between the epiglottis and the midpoint of the vocal cord. Sonographic measurement of PE/E-VC ratio.

Demographic data (age, height, body weight), ASA risk class, body mass index (BMI), Mallampati classification, mouth opening, thyromental and sternomental distance, as well as neck circumference, were recorded during the preanesthetic evaluation. Measuring for mouth opening and neck circumference were taken in the sitting position using a tape measure. Thyromental and sternomental distances were measured with a tape measure while the subjects were in the supine position with their heads in full extension. Sonographic measurements were taken for all patients in the preoperative waiting room. Ultrasonographic measurements were performed using the Esaote MyLab Five ultrasound device (MyLab Five Ultrasound System/The Netherlands). Measurements were conducted by a radiologist (HA) with 15 years of experience. Measurements were taken in both the neutral position of the patient’s head and the supine position. The measurements included TV (Figure 2), hyomental distance (Figure 3), ANS thickness at different levels (hyoid bone level, vocal cord level, thyrohyoid membrane level, thyroid isthmus level, suprasternal notch level), and the ratio of PE/E-VC using ultrasound. The recorded results are presented in Figure 4.

All patients were ventilated and intubated by the same anesthetist (SA) with five years of experience. Anesthesia induction was provided with 2–2.5 mg/kg propofol based on total body weight, 1.2 mg/kg rocuronium based on ideal body weight, and 2 μg/kg fentanyl. All patients were appropriately preoxygenated before the induction of anesthesia. They were placed in a ramp position prior to anesthesia induction. Intubation was performed directly without video laryngoscopy for all patients. The anesthetist performing mask ventilation and intubation was blind to the ultrasound measurements. Ventilation was started with a size 4 or 5 mask suitable for the patients’ the facial anatomy. The evaluation of difficult mask ventilation yielded the following results: 1) Despite applying positive pressure and 100% O2 during mask ventilation, the anesthetist alone could not raise the patient’s peripheral oxygen saturation (SpO2) above 92%, 2) the use of an oral airway or other assistive devices during the mask stage, and 3) the requirement for either holding the mask with two hands or the assistance of a second person (5). The Cormack-Lehane laryngoscopy view was used as a criterion to evaluate intubation difficulty. In the evaluation of Cormack-Lehane (C-L) laryngoscopy, C-L 1 and 2 were considered indicative of easy intubation, while and C-L 3 and 4 were indicative of difficult intubation.

Each patient was evaluated with the intubation difficulty scale (IDS) developed by Adnet et al [12]. The intubation difficulty scale score for each patient was recorded. An IDS score of 0 indicated easy intubation, a score of 1–5 denoted slight difficulty intubation, and a score above 5 indicated moderate to major difficult intubation. Impossible intubation is expressed as infinity.

### 2.1. Statistical analysis

While evaluating the data obtained from the study, statistical analysis was conducted using the SPSS 22 software (IBM SPSS Statistics, IBM Corporation, Armonk, NY, USA) package and MedCalc 19.6.1 software (MedCalc-version 19.6.1, MedCalc Software Ltd, Ostend, Belgium) package. In the analysis of categorical data, the chi-square test was used. The conformity of continuous variables to normal distribution was analyzed by the Kolmogorov-Smirnov distribution test, and the Mann-Whitney U test was applied to variables that were not normally distributed. To evaluate the predictive role of parameters related to difficult intubation or difficult mask ventilation, receiver operating characteristic (ROC) curves were generated, and cut-off values were determined using the Youden index.

In all analyzes, the results were evaluated at the significance levels of p < 0.05 and p < 0.01, with a 95% confidence interval.

## 3. Results

The number of patients in our study was determined through a power analysis based on a pilot study. Our aim was to achieve a statistical power of 90% (1–beta error (0.10)) with an alpha error of 0.05, which indicated a minimum requirement of 104 patients. To ensure the robustness of the study and account for potential dropouts or incomplete data, we decided to include a total of 120 patients.

The study was conducted between March 2020 and November 2020. During this period, we recruited and completed the necessary data collection from the selected patient cohort. By including 120 patients, we aimed to enhance the reliability and generalizability of our findings.

A total of 120 patients, comprising 74 women and 46 men, were included in the study. The COVID-19 tests for all patients returned negative results. The mean age of the study participants was found to be 34.29 ± 10.02 years. Patients with C-L 3 and 4 were included in the study as criteria for difficult intubation. The C-L grades evaluated at the intubation stage of the patients were as follows: 51 patients with C-L 1, 46 patients with C-L 2, and 23 patients with C-L 3 were observed. No patient with C-L 4 was identified. Consequently, 23 patients were assessed as having difficult intubation. Additionally, difficulty in mask ventilation was observed in 18 patients.

### 3.1. Difficult intubation

The mean age of patients in the difficult intubation group was 37.70 ± 8.38 years, while in the easy intubation group, it was 33.48 ± 10.25 years. In our study, a statistically significant increase was observed in the incidence of difficult intubation with increasing age (p = 0.027).

While 15 (65.2%) of 23 patients with difficult intubation were male, 8 (34.8%) were female. The male sex was found to be statistically significant for difficult intubation (p = 0.003).

In the 23 patients with difficult intubation, 4 (17.4%) patients had a Mallampati score of 1, 12 (52.2%) patients had a Mallampati score of 2, 5 (21.7%) patients had a Mallampati score of 3, and 2 patients (8.7%) had a Mallampati score of 4. The intubation was easy in all 45 (100%) patients with an IDS score of 0. Of 65 patients with an IDS score of 0 < IDS ≤ 5, 52 (80%) had easy intubation, while 13 (20%) had difficult intubation. All 10 (100%) patients with an IDS score >5 had difficult intubation. The increase in the IDS score was statistically significant for difficult intubation (p < 0.001) (Table 1).

ROC analysis was performed to evaluate the predictive role of classical parameters measured for difficult intubation. A mouth opening of 5 cm or less was found to be significant, with 82.7% sensitivity, 55.7% specificity, and 0.688 area under the curve (p = 0.002).

Thyromental distance of 8 cm or less was found to be significant, with 78.3% sensitivity, 46.4% specificity, and 0.641 area under the curve (p = 0.032). A neck circumference over 46 cm was found to be significant, with a sensitivity of 78.3%, a specificity of 57.7%, and an area under the curve of 0.675 (p = 0.003) (Table 2).

**Figure 5:**
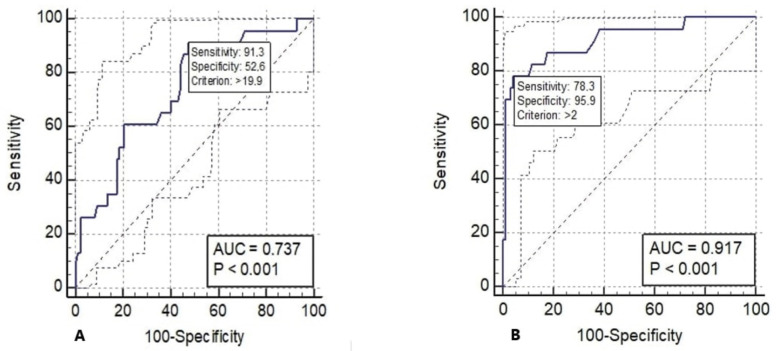
ROC analysis for difficult intubation. A) ROC analysis of preepiglottic ANS thickness value at the thyrohyoid membrane level. B) ROC analysis of PE/E-VC ratio

ROC analysis was performed to evaluate the predictive role of parameters measured by ultrasonography for difficult intubation. The cut-off values for significant parameters, the area under the curve at the 95% confidence interval, sensitivity, specificity, and p values were determined (Tables 3 and 4). The measurement of anterior neck soft tissue thickness over 19.9 mm in the preepiglottic area, measured at the level of the thyrohyoid membrane, was found to be significant with 91.3% sensitivity, 52.6% specificity, and 0.737 area under the curve (p < 0.001) (Figure 5A). A PE/E-VC above 2 was determined to be significant for difficult intubation, with a sensitivity of 78.3%, a specificity of 95.9%, and an area under the curve of 0.917 (p < 0.001) (Figure 5B).

### 3.2. Difficult mask ventilation

Difficult mask ventilation was encountered in 18 (15%) of the patients included in the study. The mean age of patients with difficult mask ventilation was 41.67 ± 9.54, while the mean age of patients with easy mask ventilation was 32.99 ± 9.58 years (p < 0.001). While the mean BMI of patients with difficult mask ventilation was 48.02±6.70 kg/m^2^, the mean BMI for patients with easy mask ventilation was 43.52 ± 4.68 kg/m^2^. The increase in BMI was found to be significant for difficult mask ventilation (p = 0.005).

While 15 (83.3%) of 18 patients with difficult mask ventilation were male, three patients (16.7%) were female; this difference was statistically significant (p < 0.001). Out of 18 patients with difficult mask ventilation, five (27.8%) patient’s Mallampati score was 1, six (33.3%) patient’s Mallampati score was 2, five (27.8%) patient’s Mallampati score was 3, and two (11.1%) patient’s Mallampati score was 4 (p = 0.040) (Table 5).

ROC analysis was performed to evaluate the predictive role of classical parameters measured for difficult mask ventilation. A mouth opening of 4.5 cm or less was found to be significant for difficult mask ventilation, with a sensitivity of 50.0%, a specificity of 76.5%, and an area under the curve of 0.688 (p = 0.002). Neck circumference above 49.5 cm was found to be significant for difficult mask ventilation, with a sensitivity of 94.4%, a specificity of 71.6%, and an area under the curve of 0.868 (p < 0.001) (Table 6).

ROC analysis was performed to evaluate the predictive role of parameters measured by ultrasonography in difficult mask ventilation. Cut-off values of the parameters, area under the curve at 95% confidence interval, sensitivity, specificity, and p values were determined (Tables 7 and 8).

**Figure 6:**
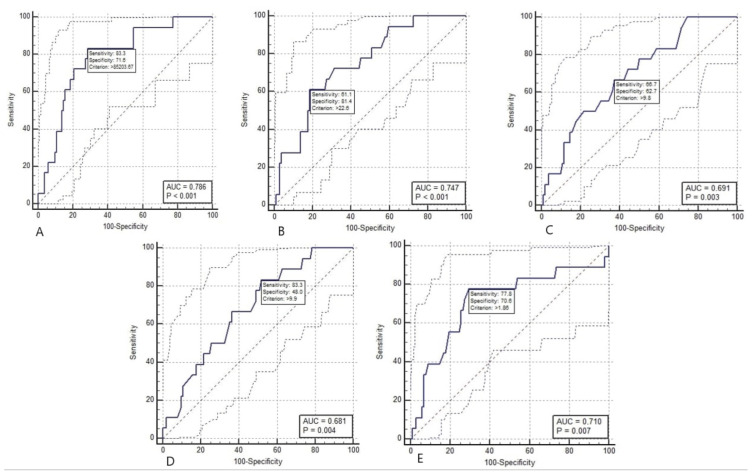
ROC analysis for difficult ventilation. A) ROC analysis of tongue volume. B) ROC analysis of preepiglottic ANS thickness at the thyrohyoid membrane level. C) ROC analysis of ANS thickness at the thyroid isthmus level. D) ROC analysis for ANS thickness value at suprasternal notch level. E) ROC analysis of PE/E-VC ratio

The measurement of tongue volume over 85,203.67 mL^3^ was found to be significant with 83.3% sensitivity, 71.6% specificity, and 0.786 area under the curve (p < 0.001) (Figure 6A).

An anterior neck soft tissue thickness greater than 22.6 mm in the preepiglottic area, measured at the level of the thyrohyoid membrane, was found to be significant with 61.1% sensitivity, 81.4% specificity, and 0.747 area under the curve (p < 0.001) (Figure 6B).An anterior neck soft tissue thickness over 9.8 mm, measured at the level of the thyroid isthmus, was found to be significant with 66.7% sensitivity, 62.7% specificity, and 0.691 area under the curve (p = 0.002) (Figure 6C).

An anterior neck soft tissue thickness over 9.9 mm, measured at the suprasternal notch level, was found to be significant with 83.3% sensitivity, 48.0% specificity, and 0.681 area under the curve (p = 0.004) (Figure 6D).

PE/E-VC ratio over 1.86 was found to be significant for difficult mask ventilation with 77.8% sensitivity, 70.6% specificity and 0.710 area under the curve (p = 0.005) (Figure 6E).

## 4. Discussion

Difficult mask ventilation and difficult intubation are more frequently encountered in obese patients. Ultrasound is a reliable and noninvasive method for the evaluation of the airway. The aim of this study was to investigate the contribution and availability of ANS thickness at different levels, TV, HMD, the ratio of PE/E-VC measured by ultrasonography for predicting difficult airway in morbidly obese patients. According to the findings of our study, ANS thickness at the level of the thyrohyoid membrane and PE/E-VC can be used for the prediction of difficult intubation. In addition, TV, preepiglottic ANS thickness at the thyrohyoid membrane level, ANS thickness at the thyroid isthmus level, ANS-suprasternal notch thickness, and PE/E-VC can be used to predict difficult mask ventilation.

Waleed et al. [13] found that, in their study conducted with morbidly obese patients, the frequency of difficult mask ventilation was 11%, and that of difficult intubation was 13%. In our study, no significant relationship was found between difficult intubation and BMI in obese patients. However, a significant relationship was found between BMI and difficult mask ventilation (p = 0.005). As the BMI increased, the difficulty in ventilation also increased.

In some studies, male sex was found to be an independent risk factor for difficult mask ventilation in morbidly obese patients [13,14]. Our study supported these findings, and the incidence of difficult mask ventilation was found to be higher in males.

There are many studies in the literature examining the risk factors for difficult mask ventilation. The incidence of difficult mask ventilation varies between 0.08% and 15% in the literature, depending on the differences in definition [15,16]. In our study, the incidence of difficult mask ventilation in obese patients was found to be 15%. This may be because only morbidly obese patients were included in our study. Another reason may be that the rate of male patients in our study was relatively higher than in some other studies.

Since the tests used to predict difficult intubation are at times insufficient, studies on tests that are easy-to-apply, practical, and highly reliable are continuing [7,9]. In some studies, ultrasonography has proven to be a reliable and noninvasive method in the evaluation of the airway [11,17,18]. Some authors have used ultrasound to predict difficult intubation, but there is currently little evidence regarding which ultrasound assessments are the best predictors. These authors have obtained measurements of different levels of cervical anterior soft tissue thickness [19,20,21]. Ultrasound has been used to assess various aspects, including tongue size [20,22], volume, floor of mouth muscles [20], preepiglottic distance [23], distance between epiglottis and vocal cords [24], and ANS thickness [19,22]. The inability to visualize the hyoid on sublingual sonography has been associated with difficult intubation [17].

Ezri et al. [19] found that the ANS thickness, measured at the level of the vocal cords in obese patients, differed significantly between those with easy and difficult intubation. However, in our study, ANS thickness measurement at the level of the vocal cords was not found to be significant for both difficult mask ventilation and difficult intubation. Ezri et al. had calculated the average of the measurements at the vocal cord level from three different locations, whereas our study involved only one measurement. Macroglossia is known as a clinical predictor for difficult airway as it does not allow the clear visualization of the larynx during direct laryngoscopy. In our study, tongue volume measurement was performed concerning both difficult mask ventilation and difficult intubation. No significant relationship was found between tongue volume and difficult intubation. However, tongue volume was found to be significant with 83.3% sensitivity and 71.6% specificity in difficult mask ventilation. HMD has been defined as a distinguishing marker in obese patients with both easy and difficult laryngoscopy [20]. In our study, HMD measurements did not show a significant difference between the difficult intubation group and the easy intubation group.

In the studies performed, the sensitivity for PE/E-VC ranged from 82% to 87.5%, and the specificity ranged from 30% to 80% [25,26]. Reddy et al. [23] concluded in their study that PE/E-VC is not an indicator of difficult intubation. Gupta et al. showed a strong correlation between PE/E-VC and Cormack-Lehane grading [24]. In our study, PE/E-VC was found to be a predictor for both difficult mask ventilation and difficult intubation. A strong correlation was found between Cormack-Lehane grade and PE/E-VC.

Geetha et al. showed that ANS thickness at the thyrohyoid membrane and vocal cord level are independent predictors for difficult intubation (27). Saru et al. used combined sonographic parameters to predict difficult intubation. In our study, ultrasound parameters were used not only to determine difficult intubation but also to evaluate difficult mask ventilation (28).

### 4.1. Limitations

The limitations of the study are that it was conducted in a single center and with a low number of patients. The study did not examine the smoking history of the patients. Furthermore, there were no grade IV Cormack-Lehane patients in the present study. Another limitation of the study is that sonographic measurements were performed by an experienced radiologist. However, it is important to note that the results might vary if these measurements were performed by different anesthetists with less experience in sonography.

### 4.2. Conclusion

In conclusion, we investigated the role of tongue volume, anterior neck soft tissue measurement, HMD and PE/E-VC ratio in predicting difficult airway by ultrasonography. Tongue volume, preepiglottic ANS thickness at the level of the thyrohyoid membrane, ANS thickness at the thyroid isthmus level, ANS thickness at the suprasternal level, and PE/E-VC ratio were found to be successful predictors of difficult mask ventilation. Preepiglottic ANS at the thyrohyoid membrane level and PE/E-VC ratio were successful in predicting difficult intubation. However, more studies are needed to explore the use of ultrasound in the evaluation of upper airways for estimating difficult airway.

## Figures and Tables

**Table 1:** Demographic data of patients for difficult intubation.

	Intubation			p

	Easy (n = 97) Cormack-Lehane 1 and 2	Difficult (n = 23) Cormack-Lehane 3	Total (n = 120)	

Age (year)	33.48 ± 10.25	37.70 ± 8.38	34.29 ± 10.02	**0.027** * m

Length (m)	1.66 ± 0.09	1.71 ± 0.07	1.67 ± 0.09	**0.015** * m

Weight (kg)	122.82 ± 19.49	131.52 ± 22.90	124.49 ± 20.38	0.093^m^

BMI (kg/m^2^)	44.13 ± 5.26	44.47 ± 5.34	44.20 ± 5.26	0.575^m^

Sex				**0.003** * k
□ Female, n (%)	66 (68.0)	8 (34.8)	74 (61.7)	
□ Male, n (%)	31 (32.0)	15 (65.2)	46 (38.3)	

Mallampati				0.185^k^
□ 1, n (%)	30 (30.9)	4 (17.4)	34 (28.3)	
□ 2, n (%)	53 (54.6)	12 (52.2)	65 (54.2)	
□ 3, n (%)	12 (12.4)	5 (21.7)	17 (14.2)	
□ 4, n (%)	2 (2.1)	2 (8.7)	4 (3.3)	

Intubation difficulty scale				**<0.001** * k
□ Easy intubation, n (%)	45 (46.4)	0 (0.0)	45 (37.5)	
□ Slight difficulty, n (%)	52 (53.6)	13 (56.5)	65 (54.2)	
□ Moderate-major, n (%)	0 (0.0)	10 (43.5)	10 (8.3)	

m Mann-Whitney U test,

k chi-square test,

* p < 0.05,

BMI = body mass index.

**Table 2:** ROC analysis of classical parameters for difficult intubation.

Parameter	Cut-off	AUC (95% CI)	Sensitivity (%)	Specificity (%)	p
Mouth opening (cm)	**≤5**	0.688 (0.597–0.770)	82.7	55.7	**0.002** *
Thyromental distance (cm)	**≤8**	0.641 (0.548–0.726)	78.3	46.4	**0.032** *
Sternomental distance (cm)	≤12.5	0.524 (0.481–0.664)	26.1	89.7	0.28
Neck circumference (cm)	**>46**	0.675 (0.583–0.757)	78.3	57.7	**0.003** *

AUC = area under the curve,

* p < 0.05,

CI = confidence interval.

**Table 3:** Statistical comparison of sonographic parameters of patients with easy and difficult intubation.

	Intubation		p
	Easy (97) mean ± standard deviation (min–max)	Difficult (23) mean ± standard deviation (min–max)	
Tongue volume (mL^3^)	77924.06 ± 18596.49 (47419.29–118981.20)	81873.13 ± 16854.57 (49238.43–108839.30)	0.273
ANS-vocal cord level (mm)	8.19 ± 1.22 (4.7–11.1)	8.61 ± 1.33 (6.2–11.6)	0.252
ANS-hyoid bone level (mm)	12.04 ± 2.58 (4.9–17.5)	12.64 ± 2.56 (8.8–17.2)	0.356
ANS-thyrohyoid membrane level (mm)	20.00 ± 3.31 (12.5–28.5)	23.13 ± 3.67 (15.0–30.4)	**<0.001** *
ANS-thyroid isthmus level (mm)	9.44 ± 2.13 (4.4–15.1)	10.16 ± 2.46 (5.0–15.2)	0.172
ANS-suprasternal notch level (mm)	10.41 ± 2.36 (3.9–17.2)	11.45 ± 2.78 (5.9–16.9)	0.096
HMD (mm)	43.45 ± 6.13 (27.9–60.1)	43.97 ± 6.44 (31.9–57.5)	0.65
PE/E-VC	1.71 ± 0.20 (1.33–2.44)	2.18 ± 0.26 (1.59–2.62)	**<0.001** *

ANS = anterior neck soft tissue, HMD = hyomental distance, PE/E-VC = the ratio of preepiglottic distance to distance between the epiglottis and the midpoint of vocal cords,

* p < 0.05.

**Table 4:** ROC analysis of predictive parameters measured by ultrasonography for difficult intubation (cut-off value, area under the curve at 95% confidence interval, sensitivity, and specificity values).

Parameter	Cut-off	AUC (95% CI)	Sensitivity (%)	Specificity (%)
Tongue volume (mL^3^)	**>**81145	0.574 (0.480–0.664)	60.9	59.8
ANS-vocal cord level (mm)	>8.9	0.577 (0.483–0.667)	43.5	75.3
ANS-hyoid bone level (mm)	>11.9	0.562 (0.469–0.652)	65.2	49.5
ANS- thyrohyoid membrane level (mm)	**>19.9**	0.737 (0.649–0.813)	91.3	52.6
ANS-thyroid isthmus level (mm)	>9.7	0.592 (0.498–0.681)	56.5	59.8
ANS-suprasternal notch level (mm)	>10.7	0.612 (0.519–0.699)	56.5	62.9
HMD (mm)	>41	0.535 (0.441–0.626)	73.9	42.3
PE/E-VC	**>2**	0.917 (0.853–0.960)	78.3	95.9

ROC = receiver operating characteristic, ANS = anterior neck soft tissue, HMD: hyomental distance, PE/E-VC = the ratio of preepiglottic distance to distance between the epiglottis and the midpoint of vocal cords, AUC = area under the curve, CI = confidence interval.

**Table 5:** Demographic data of patients with difficult mask ventilation.

	Mask ventilation			p

	Easy (102)	Difficult (18)	Total (n = 120)	

Age (year)	32.99 ± 9.58	41.67 ± 9.54	34.29 ± 1.02	**0.001** * m

Length (m)	1.66 ± 0.08	1.72 ± 0.09	1.67 ± 0.09	**0.011** * m

Weight (kg)	121.47 ± 19.30 (90–183)	141.61 ± 18.12 (110–182)	124.49 ± 20.38 (90–183)	**<0.001** * m

BMI (kg/m^2^)	43.52 ± 4.68	48.02 ± 6.70	44.20 ± 5.26	**0.005** *

Sex				**<0.001** * k
□ Female, n (%)	71 (69.6)	3 (16.7)	74 (61.7)	
□ Male, n (%)	31 (30.4)	15 (83.3)	46 (38.3)	

Mallampati				**0.040** * k
□ 1, n (%)	29 (28.4)	5 (27.8)	34 (28.3)	
□ 2, n (%)	59 (57.8)	6 (33.3)	65 (54.2)	
□ 3, n (%)	12 (11.8)	5 (27.8)	17 (14.2)	
□ 4, n (%)	2 (2.0)	2 (11.1)	4 (3.3)	

m Mann-Whitney U test,

k chi-square test,

* p < 0.05,

BMI = body mass index.

**Table 6:** ROC analysis of classical parameters for difficult mask ventilation.

Parameter	Cut-off	AUC (95% CI)	Sensitivity (%)	Specificity (%)	p
Mouth opening (cm)	**≤4.5**	0.688(0.597–0.769)	50.0	76.5	**0.002** *
Thyromental distance (cm)	≤7	0.663 (0.571–0.747)	61.1	73.6	0.052
Sternomental distance (cm)	≤15	0.615 (0.522–0.703)	83.3	36.3	0.104
Neck circumference (cm)	**>49.5**	0.868 (0.794–0.923)	94.4	71.6	**<0.001** *

ROC = receiver operating characteristic, AUC = area under the curve,

* p < 0.05,

CI = confidence interval.

**Table 7:** Statistical comparison of sonographic parameters of patients with easy and difficult ventilation.

	Ventilation		p
	Easy (102) mean ± standard deviation (min–max)	Difficult (18) mean ± standard deviation (min–max)	
Tongue volume (mL^3^)	76002.14 ± 17542.57 (47419.29–117021.45)	93860.94 ± 14906.75 (59122.16–118981.20)	**<0.001** *
ANS-vocal cord level (mm)	8.29 ± 1.26 (4.7–11.6)	8.12 ± 1.16 (5.4–10.5)	0.503
ANS-hyoid bone level (mm)	11.99 ± 2.60 (4.9–17.5)	13.08 ± 2.27 (8.8–16.7)	0.084
ANS-thyrohyoid membrane level (mm)	20.13 ± 3.46 (12.5–30.4)	23.27 ± 3.15 (18.4–29.6)	**0.001** *
ANS-thyroid isthmus level (mm)	9.35 ± 2.15 (4.4–15.2)	10.86 ± 2.11 (8.2–15.1)	**0.010** *
ANS-suprasternal notch level (mm)	10.38 ± 2.44 (3.9–17.1)	11.89 ± 2.32 (8.6–17.2)	**0.014** *
HMD (mm)	43.35 ± 6.02 (27.9–60.1)	44.70 ± 7.06 (31.9–58.1)	0.362
PE/E-VC	1.77 ± 0.26 (1.35–2.62)	1.98 ± 0.34 (1.33–2.60)	**0.005** *

ANS = anterior neck soft tissue, HMD = hyomental distance, PE/E-VC = the ratio of preepiglottic distance to distance between the epiglottis and the midpoint of vocal cords,

* p < 0.05.

**Table 8:** ROC analysis of predictive parameters measured by ultrasonography for difficult mask ventilation (cut-off value, area under the curve at 95% confidence interval, sensitivity, and specificity values).

Parameter	Cut-off	AUC (95% CI)	Sensitivity (%)	Specificity (%)
Tongue volume (mL^3^)	**>85203.67**	0.786 (0.702–0.856)	83.3	71.6
ANS- vocal cord level (mm)	≤8	0.550 (0.456–0.641)	61.1	58.8
ANS-hyoid bone level (mm)	>12.4	0.628 (0.535–0.714)	72.2	58.8
ANS-thyrohyoid membrane level (mm)	**>22.6**	0.747 (0.660–0.822)	61.1	81.4
ANS-thyroid isthmus level (mm)	**>9.8**	0.691 (0.600–0.772)	66.7	62.7
ANS-suprasternal notch level (mm)	**>9.9**	0.681 (0.590–0.763)	83.3	48.0
HMD (mm)	>41	0.568 (0.474–0.658)	77.8	42.2
PE/E-VC	**>1.86**	0.710 (0.620–0.789)	77.8	70.6

ROC = receiver operating characteristic, ANS = anterior neck soft tissue, HMD = hyomental distance, PE/E-VC = the ratio of preepiglottic distance to distance between the epiglottis and the midpoint of vocal cords, AUC = area under the curve, CI = confidence interval.
